# Potential strategies for strengthening surveillance of lymphatic filariasis in American Samoa after mass drug administration: Reducing ‘number needed to test’ by targeting older age groups, hotspots, and household members of infected persons

**DOI:** 10.1371/journal.pntd.0008916

**Published:** 2020-12-28

**Authors:** Colleen L. Lau, Meru Sheel, Katherine Gass, Saipale Fuimaono, Michael C. David, Kimberly Y. Won, Sarah Sheridan, Patricia M. Graves

**Affiliations:** 1 Research School of Population Health, Australian National University, Canberra, Australia; 2 Neglected Tropical Diseases Support Center, Task Force for Global Heath, Decatur, Georgia, United States of America; 3 American Samoa Department of Health, Pago Pago, American Samoa; 4 School of Medicine and Public Health, The University of Newcastle, Gosford, Australia; 5 Centers for Disease Control and Prevention, Division of Parasitic Diseases and Malaria, Atlanta, Georgia, United States of America; 6 School of Public Health and Community Medicine, University of New South Wales, Sydney, Australia; 7 College of Public Health, Medical and Veterinary Sciences, James Cook University, Cairns, Australia; University of Zurich, SWITZERLAND

## Abstract

Under the Global Programme to Eliminate Lymphatic Filariasis (LF), American Samoa conducted mass drug administration (MDA) from 2000–2006. Despite passing Transmission Assessment Surveys (TAS) in 2011/2012 and 2015, American Samoa failed TAS-3 in 2016, with antigen (Ag) prevalence of 0.7% (95%CI 0.3–1.8%) in 6–7 year-olds. A 2016 community survey (Ag prevalence 6.2% (95%CI 4.4–8.5%) in age ≥8 years) confirmed resurgence. Using data from the 2016 survey, this study aims to i) investigate antibody prevalence in TAS-3 and the community survey, ii) identify risk factors associated with being seropositive for Ag and anti-filarial antibodies, and iii) compare the efficiency of different sampling strategies for identifying seropositive persons in the post-MDA setting. Antibody prevalence in TAS-3 (n = 1143) were 1.6% for Bm14 (95%CI 0.9–2.9%), 7.9% for Wb123 (95%CI 6.4–9.6%), and 20.2% for Bm33 (95%CI 16.7–24.3%); and in the community survey (n = 2507), 13.9% for Bm14 (95%CI 11.2–17.2%), 27.9% for Wb123 (95%CI 24.6–31.4%), and 47.3% for Bm33 (95%CI 42.1–52.6%). Multivariable logistic regression was used to identify risk factors for being seropositive for Ag and antibodies. Higher Ag prevalence was found in males (adjusted odds ratio [aOR] 3.01), age ≥18 years (aOR 2.18), residents of Fagali’i (aOR 15.81), and outdoor workers (aOR 2.61). Ag prevalence was 20.7% (95%CI 9.7–53.5%) in households of Ag-positive children identified in TAS-3. We used NNTest^av^ (average number needed to test to identify one positive) to compare the efficiency of the following strategies for identifying persons who were seropositive for Ag and each antibody: i) TAS of 6–7 year-old children, ii) population representative surveys of older age groups, and iii) targeted surveillance of subpopulations at higher risk of being seropositive (older ages, householders of Ag-positive TAS children, and known hotspots). For Ag, NNTest^av^ ranged from 142.5 for TAS, to <5 for households of index children. NNTest^av^ was lower in older ages, and highest for Ag, followed by Bm14, Wb123 and Bm33 antibodies. We propose a multi-stage surveillance strategy, starting with population-representative sampling (e.g. TAS or population representative survey of older ages), followed by strategies that target subpopulations and/or locations with low NNTest^av^. This approach could potentially improve the efficiency of identifying remaining infected persons and residual hotspots. Surveillance programs should also explore the utility of antibodies as indicators of transmission.

## Author summary

Lymphatic filariasis (LF) is a parasitic infection transmitted by mosquito bites. Globally, tens of millions are infected, with many disfigured and disabled by severe damage to their lymphatic systems, such as severe swelling of the legs (elephantiasis) or scrotum (hydrocele). The Global Programme to Eliminate LF (GPELF) aims to interrupt disease transmission through mass drug administration (MDA), and to control illness and suffering in affected persons. The World Health Organization recommends conducting Transmission Assessment Surveys (TAS) in school children aged 6 to 7 years to determine if infection rates have dropped to levels where disease transmission is no longer sustainable. From 2000–2006, American Samoa conducted MDA and made significant progress towards eliminating LF. However, despite passing TAS in 2011/2012 and 2015, surveys in 2016 showed evidence of resurgence. This study aimed to investigate the prevalence of anti-filarial antibodies in American Samoa in 2016; identify risk factors for testing positive for antigen, microfilaria and antibodies; and compare the efficiency of different sampling strategies for identifying persons who test positive. The sampling strategies that we compared included testing of 6–7 year-old children, population representative surveys of older age groups, and targeted surveys of high-risk groups such as older people, household members of infected children identified through TAS, and known hotspots. Based on our findings, we recommended that in addition to TAS, strategies that target high-risk populations and hotspots would strengthen surveillance and help countries achieve their goals of LF elimination.

## Introduction

Lymphatic filariasis (LF) is a mosquito-borne parasitic disease caused by *Wuchereria bancrofti* and *Brugia* species. The Global Programme to Eliminate Lymphatic Filariasis (GPELF), established by the World Health Organization (WHO) in 2000, aims to eliminate the disease as a public health problem through annual mass drug administration (MDA) and alleviation of morbidity and disability in affected persons. Since 2000, GPELF has made enormous progress, and by 2018, the program had delivered 7.7 billion drug treatments to >910 million people in 68 of the 72 LF-endemic countries around the world [1]. By 2019, 14 countries had been validated by WHO as having eliminated LF as a public health problem, but 893 million people in 49 countries still need MDA [1]. To determine whether transmission has reached sufficiently low levels so that MDA can be safely stopped, WHO currently recommends using Transmission Assessment Surveys (TAS), which use critical cut-off numbers of antigen (Ag) positive children aged 6–7 years in defined evaluation units to determine if prevalence has reduced to a level where transmission is thought to be no longer sustainable [2]. After passing the first TAS, repeated surveys are recommended every 2–3 years for ongoing post-MDA surveillance.

Although TAS has been a useful tool for making decisions to stop MDA and for post-MDA surveillance, there is emerging evidence that the current TAS guidelines may not be sufficiently sensitive for identifying localised areas of ongoing transmission, especially in the post-MDA setting when prevalence has reached very low levels and the geographic distribution of residual infections is highly heterogenous (e.g. localised areas where infection prevalence is significantly higher than the rest of the surveillance area). This concern has been reported by studies from diverse settings around the world including American Samoa [3–6], Samoa [7], Sri Lanka [8], India [9], and Haiti [10,11], suggesting that additional and/or alternative surveillance strategies are required to identify localised areas of residual transmission, and also provide earlier signals of any resurgence so that programmatic gains can be protected, and long-term success of the GPELF ensured.

The GPELF is one of the largest and most ambitious public health intervention programs in the world, and it is inevitable that challenges will emerge as countries progress through the program. Over the years, operational research has provided valuable evidence to inform and improve many GPELF activities, including diagnostics, surveillance, monitoring, and evaluation [12]. Currently, the WHO and GPELF have identified standardised cost-effective surveillance strategies as one of the most important operational research needs [13]. Because population-based surveys are resource intensive, potential ways of improving their cost-effectiveness include integrating post-MDA surveillance with other public health programs, and opportunistic screening of populations, e.g. during blood donation, antenatal visits, and pre-employment tests. Other approaches include more sensitive surveillance strategies, such as the detection of anti-filarial antibodies (which may provide earlier indication of transmission and recrudescence compared to Ag) [14,15], and molecular xenomonitoring (detection of filarial DNA in mosquitoes) [16–19].

American Samoa, a US territory in the South Pacific, is a group of small remote islands where LF is endemic. Infection is caused by *W*. *bancrofti* and transmitted predominantly by the highly efficient day-biting *Aedes polynesiensis*. As part of the Pacific Programme to Eliminate LF (PacELF), a baseline survey in 1999 found an Ag prevalence of 16.5% as measured by the Alere rapid immunochromatographic test (ICT) [20]. Seven annual rounds of MDA were distributed from 2000–2006 using diethylcarbamazine (DEC) and albendazole [6,21]. MDA coverage was initially poor (19% in 2000 and 49% in 2002) but improved from 2003 to 2006 (≥65%) through community mobilisation, behaviour change communication, drug delivery strategies, and involvement of churches [6,21].

American Samoa passed TAS-1 in 2011/2012 and TAS-2 in 2015; two Ag-positive children were identified in TAS-1, and one identified in TAS-2 (critical cut-off for passing TAS was a maximum of six children) [15]. Despite passing TAS, multiple research studies conducted during the same time period raised suspicions of ongoing transmission. The studies included opportunistic screening of a serum bank from a population-representative survey of adults [3], testing adults at a work place and a pre-employment clinic [4,22], testing residents in suspected hotspots [4], and molecular xenomonitoring of mosquitoes [23]. Clustering and persistence of antibody responses noted in TAS-1 and TAS-2 may also have been an indication of ongoing transmission [15].

In 2016, approximately 10 years after the last round of MDA, American Samoa failed TAS-3 [5], confirming the earlier suspicions of ongoing transmission. In hindsight, resurgence could potentially have been detected earlier using surveillance strategies other than or in addition to the standard TAS. In parallel with TAS-3 in 2016, a community-based survey of 2507 people aged ≥8 years found an Ag prevalence of 6.2% (95% CI 4.5–8.6%), further confirming resurgence [5]. The study found that the community-based survey was logistically more difficult and expensive than the school-based TAS-3, but provided more detailed epidemiological information, including localised areas of high Ag prevalence. The study also found that although the school-based survey had limited sensitivity and negative predictive value for identifying villages with ongoing transmission, specificity and positive predictive value were high [5]. In other words, although TAS may not be sufficiently sensitive for detecting all areas with ongoing transmission, the residential locations of Ag-positive school children could lead us to at least some of the reservoirs of ongoing transmission, where more targeted and/or intensive surveillance could potentially be valuable.

According to current WHO guidelines for TAS, for any given population size in an evaluation unit, both the sample size and the threshold number for “passing” are fixed. Therefore, sensitivity and cost-effectiveness of TAS for detecting residual areas of transmission will gradually erode over time as prevalence decreases. In other words, if population representative sampling is used for surveillance (e.g. the current TAS protocols), the average number of people who need to be tested in order to find one abnormal result, or ‘Number Needed to Test’ (NNTest^av^) will increase as infection prevalence decreases. The NNTest is similar in concept to the commonly used ‘Number needed to screen’ (NNS) and ‘Number needed to treat’ (NNT) measures [24,25]. The advantages of NNS and NNT measures over more traditional metrics such as relative risks and odds ratios is that they are more easily understood by clinicians and patients, and intuitively more useful for decision making. While NNS and NNT are typically used to measure the benefit of screening or treatment on a specific clinical outcome, we use NNTest^av^ here only as a measure of the probability of identifying a positive test. In this context, NNTest^av^ could be used to measure and rank the relative sensitivity and efficiency of different surveillance strategies or combinations of strategies, e.g. population representative sampling vs strategies that target high-risk populations and/or locations, using different serological markers, and in different age groups. NNTest^av^ could be used as a summary measure to compare the conditional probabilities of identifying a positive test in subgroups, e.g. positive Ag in 6–7 year-old children who live in known LF hotspots vs positive antibody in adults in randomly selected villages.

Building on our previous report of the 2016 studies in American Samoa [5], this study aims to i) investigate antibody prevalence in the 2016 TAS-3 and community survey, ii) identify risk factors associated with being seropositive for Ag, Mf and anti-filarial antibodies (Bm14, Wb123, and Bm33), and iii) use NNTest^av^ to compare the efficiency of identifying seropositive persons by using different sampling strategies (TAS of 6–7 year-old children, population representative surveys of older age groups, and targeted surveillance of subpopulations at higher risk of being seropositive).

## Methods

### Ethics statement

Written informed consent was obtained from adult participants. For participants aged <18 years, verbal assent was obtained from the child, and formal written consent was obtained from a parent or guardian. The study was conducted in collaboration with the American Samoa Department of Health, and official permissions for school and village visits were granted by the Department of Education and the Department of Samoa Affairs, respectively. All field activities were carried out in a culturally appropriate and sensitive manner with bilingual local field teams, and with verbal approval from village chiefs or mayors prior to conducting the community surveys. Surveys were conducted in English or Samoan depending on the participants’ preference. Ethics approvals were granted by American Samoa Institutional Review Board, and the Human Research Ethics Committee at the Australian National University (protocol number 2016/482). The Institutional Review Board of the U.S. Centers for Disease Control and Prevention (CDC) determined CDC to be a non-engaged research partner.

### Study location

American Samoa consists of a group of small remote islands in the South Pacific, with an estimated population of 60,200 in 2016 [26]. Over 95% of the population live on the largest island of Tutuila and the adjacent island of Aunu’u. Very small populations reside in the remote Manu’a islands, which were not included in this study.

### Survey components

**Fig 1:**
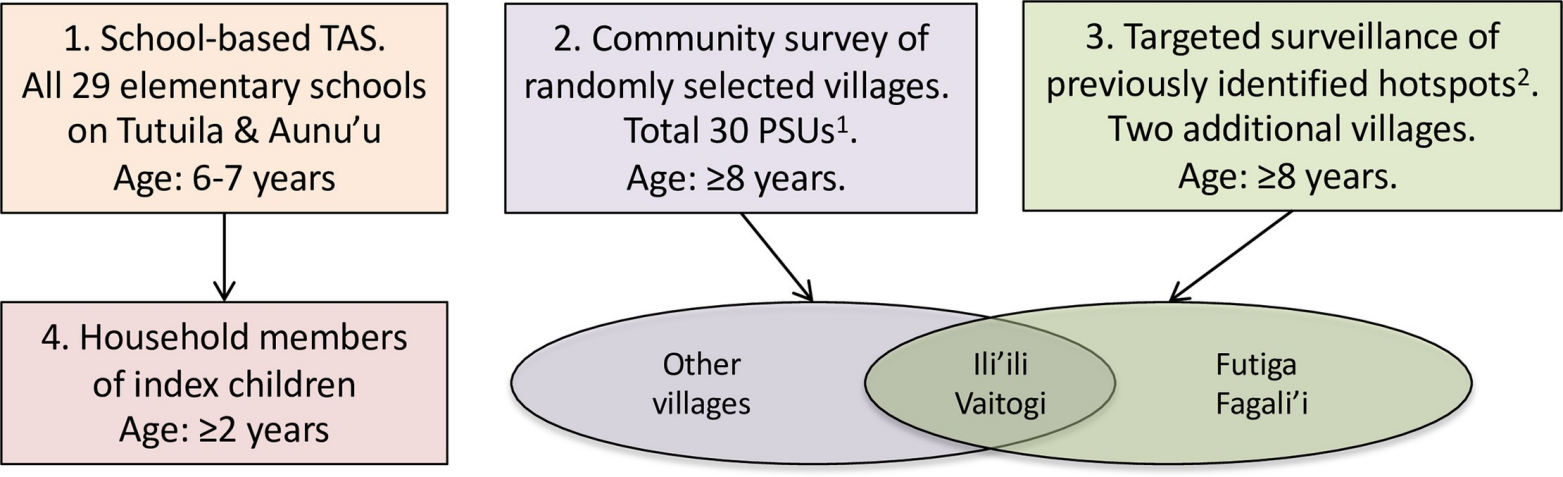
Summary of survey components, American Samoa 2016: 1) School-based TAS, 2) Community-based survey of randomly selected villages, 3) Targeted surveillance of previously identified hotspots, and 4) Household members of index children. ^1^PSU = Primary sampling unit, with maximum population of 2000. Most PSUs consisted of one village, but some of the larger villages were split into PSUs of <2000 people before random selection. The very small contiguous villages of Satala, Anua, and Atu’u were combined to form one PSU. ^2^Two previously identified hotspots were Fagali’i village and the contiguous villages of Ili’ili/Vaitogi/Futiga. Ili’ili and Vaitogi villages were randomly selected for the community survey, and the other two specifically included.

**Fig 2:**
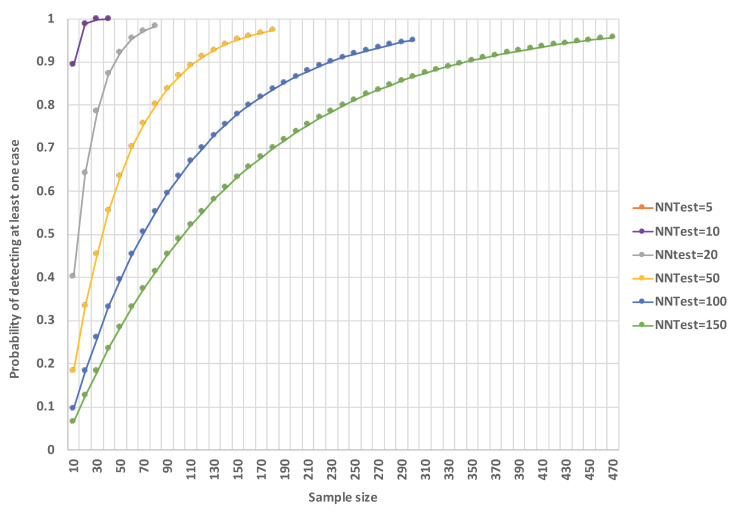
Survey locations in American Samoa 2016: elementary schools (blue circles), randomly selected villages (yellow), and additional hotspot villages (orange).

The 2016 field study in American Samoa included four components, as summarised in Figs 1 and 2 and further described below.

#### School-based TAS-3 of 6–7 year-old children

A school based TAS-3 was conducted according to WHO guidelines, and the sampling design and Ag results have been previously described [5]. Briefly, the survey invited all Grade 1 and 2 children from all 29 elementary schools on the islands of Tutuila and Aunu’u to participate, and a total of 1143 children were tested (Fig 2). Based on WHO guidelines, the critical cut-off for passing TAS-3 was a maximum of six Ag-positive children [2].

#### Community-based survey of randomly selected villages (age ≥8 years)

In parallel with TAS-3, a community-based population proportionate survey was conducted in 30 randomly selected primary sampling units (PSUs) (Fig 2). Based on WHO guidelines, PSUs were selected using a multi-stage equal probability cluster survey method and described in detail in our previous publication [5]. Most PSUs consisted of one village, but some of the larger villages were split into PSUs of <2000 people, and the very small contiguous villages of Satala, Anua, and Atu’u were combined to form one PSU. Households were randomly selected within each village, and all household members aged ≥8 years were invited to participate.

#### Targeted surveillance of previously identified hotspots

In this paper, a ‘hotspot’ is defined as an area where antigen prevalence is above WHO recommended thresholds and significantly higher than the rest of the surveillance area based on previous spatial and/or non-spatial statistical analyses. Our previous studies identified [3] and later confirmed [4] two transmission hotspots in American Samoa, located in the contiguous large villages of Ili’ili, Vaitogi and Futiga, and the small remote village of Fagali’i in the far west of Tutuila (Fig 2). The two Ag-positive children identified in TAS-1 and the one Ag-positive child identified in TAS-2 all attend the same elementary school located in the first hotspot. The villages of Ili’ili and Vaitogi were randomly selected for the 2016 community-based survey described above, and Futiga and Fagali’i were specifically added to the survey to allow further assessment of the hotspots. In the two additional villages, the survey was conducted using the same methods as the randomly selected villages. In Fagali’i, participants also included volunteers from households that were not randomly selected; demographics and infection rates did not differ significantly between volunteers and randomly selected community members, and the two groups were combined for estimates of village-level seroprevalence.

#### Household members of Ag-positive school children from TAS-3

All Ag-positive children from TAS-3 (referred to from here as ‘index children’) were followed up at home and provided with treatment with DEC and albendazole according to WHO guidelines (see below). All household members of index children who were aged ≥2 years and present during the home visits were invited to participate in the study, using the same survey methods as the community-based survey. If households had already been sampled as part of the randomly selected households for the community survey, household members aged 2–7 years and others who had not already been tested during the community survey were also invited to participate.

### Questionnaires and household locations

Standardised electronic questionnaires were used to collect demographic and household data, including gender, age, work location, time lived in American Samoa, ever taken MDA, travel in past 12 months, and living in a known hotspot. Work location was classified as indoor, outdoor, tuna cannery (largest private employer in American Samoa), and other (including mixed indoor/outdoor, unemployed, retired or unknown). For the community survey, hotspots survey, and household visits of index children, GPS locations of households were recorded. Data were collected by bilingual field research assistants (in Samoan or English according to each participant’s preference), using smartphones and the LINKS electronic database system [27].

### Blood collection and laboratory tests

The Alere Filariasis Test Strip (FTS) was used to detect circulating filarial Ag. For each participant, a 200uL finger-prick blood sample was collected into heparinised microtainers (BD, North Ryde, NSW Australia), and samples were kept cool and transported to a laboratory where testing by FTS was conducted on the same or next day. For household members of index children, samples were tested during the household visits so that Ag-positive persons could be provided with treatment at that time and avoid the need for additional visits. For Ag-positive persons, microfilaria (Mf) slides were prepared using standard procedures [2]. For all participants, dried blood spots (DBS) were prepared using TropBio filter papers (Cellabs, Sydney, Australia), and shipped to the US CDC for assays of Wb123, Bm14, and Bm33 antibodies using previously described methods [15].

### Treatment of antigen-positive persons

Unless medically contraindicated, Ag-positive individuals were offered treatment with DEC (6mg/kg) and Albendazole (400mg). As described above, Ag-positive children identified through TAS-3 were treated at home, in the presence of a parent or guardian. Ag-positive persons identified through the community survey were contacted by phone and invited (in some cases with family members) to attend a clinic for treatment. All medications were provided free of charge.

### Definition of subgroups used in analyses

**Fig 3:**
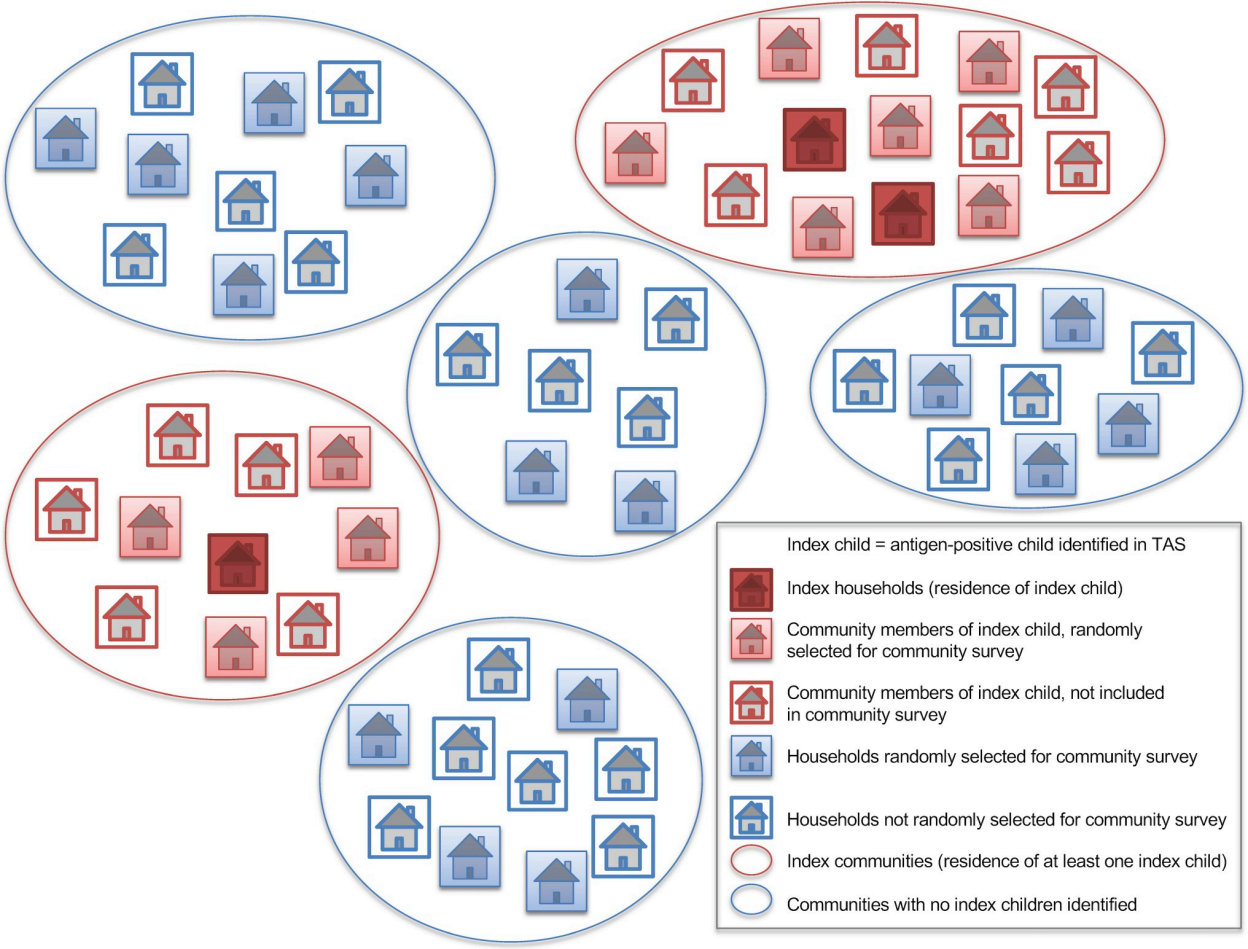
Definitions of subgroups used for analyses. An index child was defined as an antigen-positive child identified in TAS. Index households and communities were defined as those where an index child lived. These groups were compared with households and communities without an index children.

The following definitions were used for the different subgroups included in the analyses, and a schematic representation is provided in Fig 3:

- Index child–Ag-positive child identified through TAS (data from survey component 1)
- Index households–household members of index child (data from survey component 4)
- Index villages/communities–villages/communities where at least one index child lived (data from survey components 2 & 3)
- Randomly selected households–households selected for the community survey (data from survey component 2)

### Statistical analysis and mapping

Analyses were performed using Microsoft Excel (version 16.32, Microsoft Corporation, Redmond, WA) or Stata (version 14, Stata Corp, College Station, TX), and *p* values of <0.05 were considered statistically significant. Spatial data on island and village boundaries in American Samoa were obtained from the American Samoa Coastal and Marine Spatial Planning Data Portal [28]. Geographic information systems (GIS) software (ArcGIS v10.4.1, Environmental Systems Research Institute, Redlands CA) was used to manage spatial data and produce maps. A STROBE checklist for cross-sectional studies is provided in S1 Checklist.

#### Crude and adjusted seroprevalence

Crude prevalence were estimated for Ag, Mf, and antibodies (Bm14, Wb123, and Bm33), and 95% confidence intervals (CI) were calculated using the Clopper-Pearson binomial exact method [29]. Adjusted seroprevalence for Ag and antibodies (adjusted for age, sex, and survey design) were calculated using previously described methods [5]. Detailed explanation of the methods used to calculate adjusted seroprevalence are included in S1 Text, S1 Table and S2 Table.

Population representative estimates of seroprevalence for 6–7 year-olds were calculated using TAS-3 results, and for the older age groups (≥8 years) using data from the community survey of randomly selected villages. Adjusted seroprevalence of Ag and antibodies were also calculated for the following subgroups based on age, residence in the two previously known transmission hotspots, and residential proximity to an index child:

1. Age groups, based on ages that could potentially be targeted by different surveillance strategies:
  - 6–7 year-old children (e.g. routine TAS)
  - 8–12 year-old children (e.g. older elementary school)
  - 13–17 year-old children (e.g. high school)
  - ≥8 years old (e.g. the 2016 community-based survey in American Samoa)
  - ≥18 years old (e.g. surveillance of adults at a work place)
2. Residents of previously identified hotspots, stratified by age groups
  - Residents of Ili’ili, Vaitogi, Futiga (total population 5877)
  - Residents of Fagali’i (total population 247)
3. Household members of index children (limited to those aged ≥8 years to allow comparison with results from population representative community survey).

These three factors–age, residents of hotspots, and household members of index children–were specifically chosen for exploring the NNTest concept because targeted strategies could be relatively easily developed for these subgroups. In comparison, it would be logistically more difficult to develop surveillance strategies that target other risk factors such as number of years lived in American Samoa, travel history, or previous participation in MDA.

#### Risk factor analysis

We undertook descriptive analyses of demographic and behavioural data collected through questionnaires, and determined statistical differences between proportions using Pearson’s chi squared tests, Fisher’s exact tests, or two-sample test of proportions (Stata command prtesti). Logistic regression was used to assess associations between questionnaire variables and Ag, Mf, and each antibody. Any variables with p <0.2 on univariate analyses were tested using multivariable logistic regression. Variables were sequentially removed from the multivariable models to arrive at the most parsimonious models, in which variables with p <0.05 were retained.

#### Intra-cluster correlation

Intra-cluster correlation (ICC) was used to provide a measure of the degree of clustering for Ag, Mf and each Ab within PSUs and households. ICCs were estimated using a multi-level logistic regression model (Stata commands ‘melogit’ and ‘estat icc’) with age and sex included as fixed effects, and two levels: PSUs and households. ICC values range from zero to one and provide a measure of how similar observations are at each level, in this case PSUs and households. A higher ICC indicates that the outcome measure is more homogenous (i.e. high degree of clustering) at that level.

#### Number needed to test

For Ag and each antibody, three NNTest measures were calculated using the following formula, where p = adjusted seroprevalence for each subgroup), N = sample size, and probability of detecting at least one case = 1-(1-p)^N:

- NNTest^av^. Average number needed to test in order to identify one positive result: 1/p.
- NNTest^50^. Number (N) needed to test to provide a 50% chance of identifying at least one positive result, where 1-(1-p)^N = 0.5, i.e. NNTest^50^ = *ln* (0.5) / *ln* (1-p)
- NNTest^95^. Number (N) needed to test to provide a 95% chance of identifying at least one positive result, where 1-(1-p)^N = 0.95, i.e. NNTest^95^ = *ln* (0.05) / *ln* (1-p)

NNTest^av^ was not calculated for Mf because it is impractical to use Mf as a screening tool. It is important to note that NNTest^av^ itself does not provide an indication of the sample size required for surveys, and if NNTest^av^ is *n* for a specific strategy, testing *n* number of people certainly would not guarantee that an infected person will be identified. NNTest^50^ and NNTest^95^ are used here as examples of the sample size required to provide different degrees of certainty (50% and 95% certain) that positive cases are not missed.

## Results

### Seroprevalence of antigen and antibodies

#### School-based TAS-3 of 6–7 year-old children

**Fig 4:**
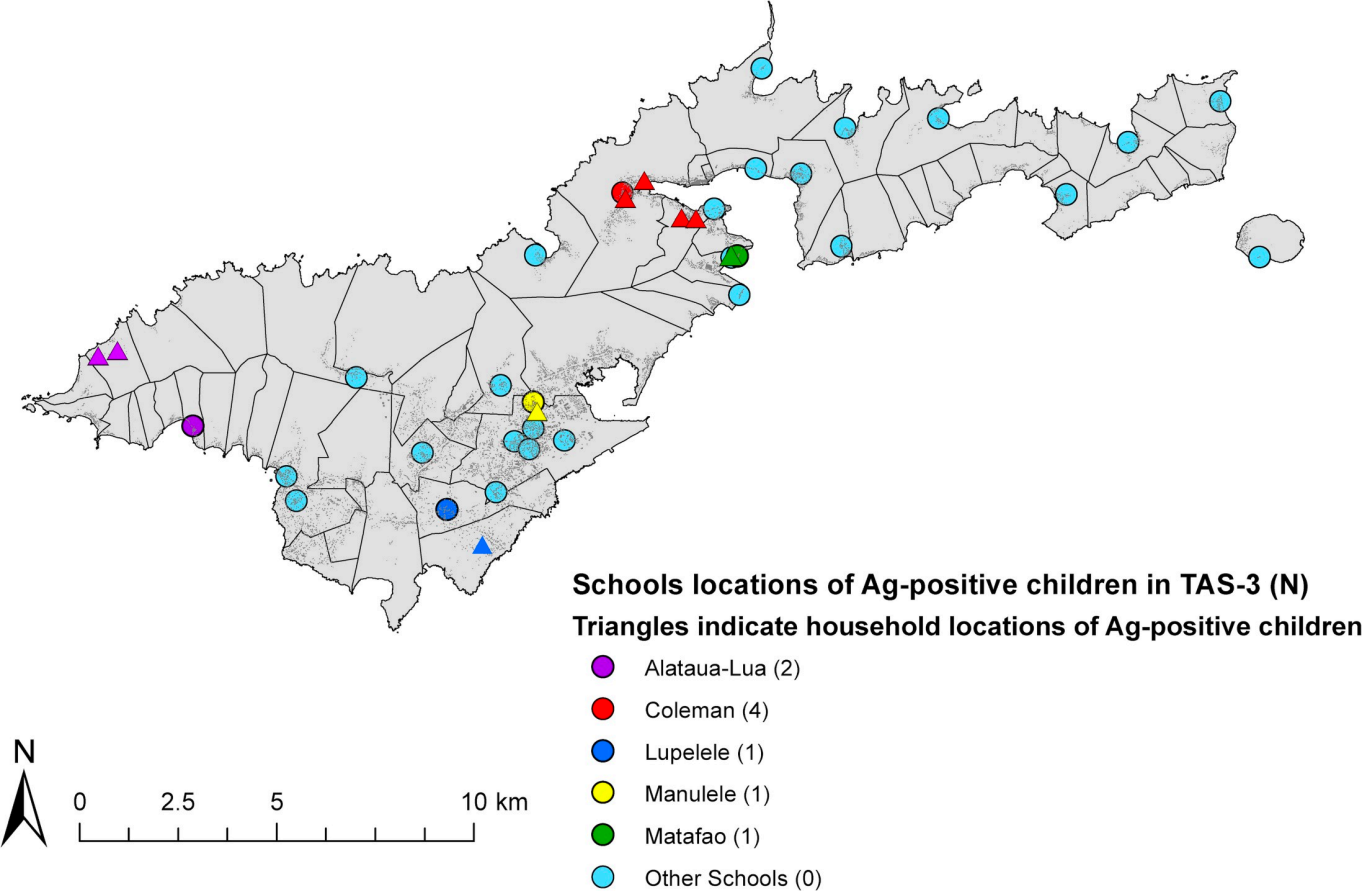
Locations of schools (circles) and households (triangles) of Ag-positive children identified in TAS-3 (index children), American Samoa 2016. Colours of triangles indicate the school location of each index child.

Antigen results from the school-based TAS-3 have been previously described in detail [5]. Briefly, of the 1143 school children tested in TAS-3, 51.2% were female, and 90.4% were aged 6–7 years old. The children represented 52.4% of all Grade 1 and 2 enrolments and were highly representative of 6–7 year-olds in American Samoa. Nine Ag-positive children (index children) were identified, and one of them was Mf-positive. After adjusting for sex and participation rates by school (average 52.4%), the estimated overall Ag prevalence was 0.7% (95% CI 0.3–1.8%) [5]. The nine index children attended five different schools and lived in six different villages (Fig 4). Overall adjusted antibody prevalence in TAS-3 were 1.6% for Bm14 (95% CI 0.9–2.9%), 7.9% for Wb123 (95% CI 6.4–9.6%), and 20.2% for Bm33 (95%CI 16.7–24.3%). Amongst the Ag-positive children, 55.6% (95% CI 21.1–86.3%) were positive for Bm14, 66.7% (95% CI 29.9–92.5%) for Wb123, and 88.9% (95% CI 51.8–99.7%) for Bm33 antibodies. Five of the nine Ag-positive children (55.6%) were seropositive for all three antibodies, while one child (11.1%) was seronegative for all antibodies. All nine Ag-positive children were followed up at home and provided with treatment.

#### Community-based survey of randomly selected villages (age ≥8 years)

**Fig 5:**
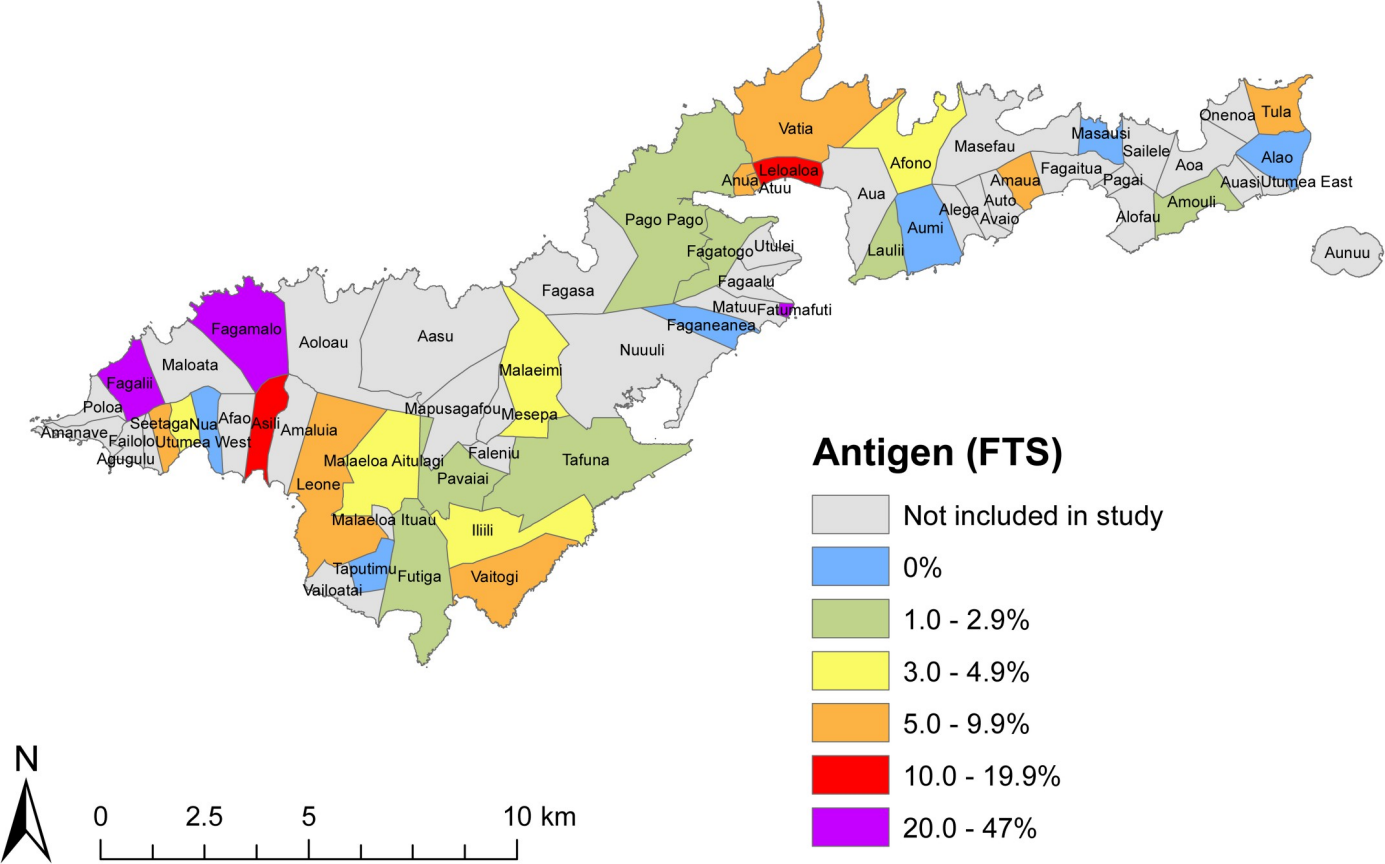
Adjusted seroprevalence by village in community survey (age ≥8 years) of randomly selected villages and previously identified hotspots, American Samoa 2016.

**Fig 6:**
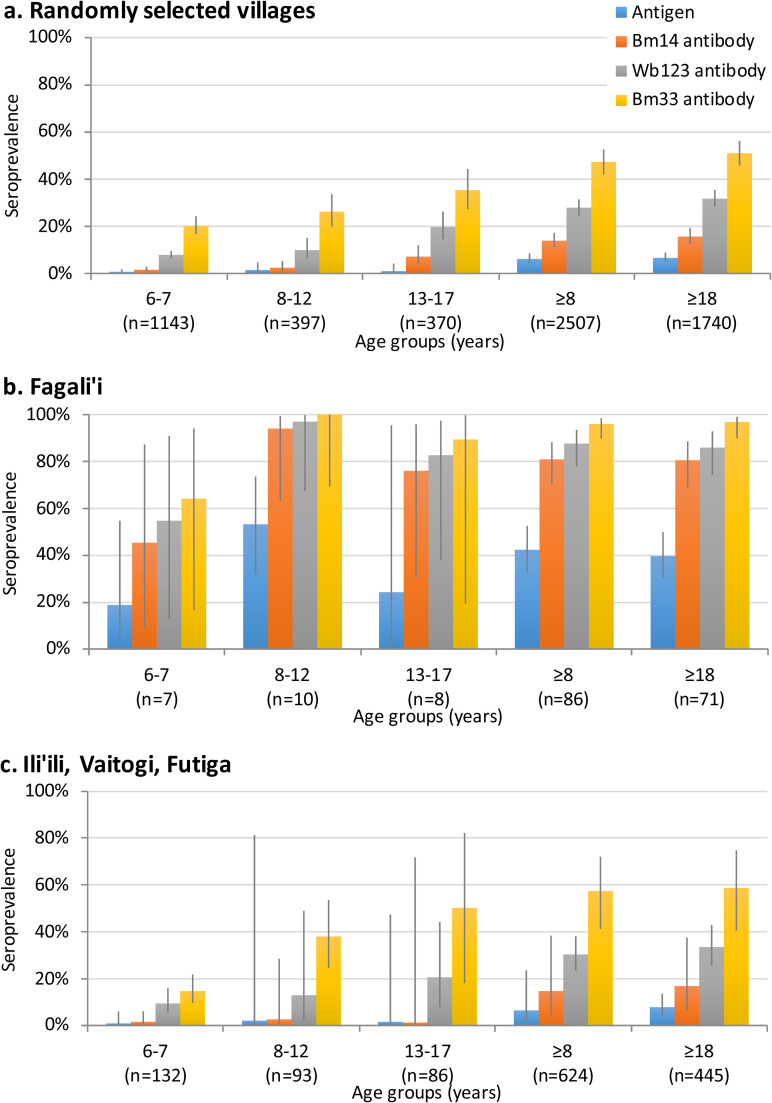
Adjusted seroprevalence (and 95% CI) of Ag and antibodies by age groups for a) randomly selected communities, b) Fagali’i, and c) Ili’ili/Vaitogi/Futiga.

The community survey of 30 randomly selected PSUs included 2507 participants aged ≥8 years, and found a total of 102 Ag-positive persons [5]. Of 86 Ag-positive community members where Mf slides were available, 22 (25.6%) were Mf-positive. After adjusting for age, sex, and the survey design, the estimated overall Ag prevalence was 6.2% (95% CI 4.4–8.5%), with village-level Ag prevalence ranging from 0% to 47% [5] (Fig 5). Overall adjusted antibody prevalence in the community survey were 13.9% for Bm14 (95% CI 11.2–17.2%), 27.9% for Wb123 (95% CI 24.6–31.4%), and 47.3% for Bm33 (95% CI 42.1–52.6%). Amongst the 101 Ag-positive persons where antibody results were available, 76.2% (95% CI 66.7–84.1%) were positive for Bm14 Ab, 86.1% (95% CI 77.8–92.2%) for Wb123 Ab, and 93.1% (95% CI 86.2–97.2%) for Bm33 Ab. The seroprevalence of Ag and antibodies varied significantly between age groups (Fig 6A).

#### Residents of previously identified hotspots

The survey in previously identified hotspots included 86 participants aged ≥8 years from Fagali’i (age 8–77 years), and 624 from Ili’ili/Vaitogi/Futiga (age 8–90 years) where Ag results were available. Of these participants, positive antigen was identified in 32 (36.8%) from Fagali’i and 36 (5.8%) from Ili’ili/Vaitogi/Futiga. Adjusted Ag prevalence for Fagali’i and Ili’ili/Vaitogi/Futiga were 42.3% (95% CI 32.7–52.5%) and 6.5% (95% CI 1.5–23.6%), respectively. Of the 27 Mf slides examined from Fagali’i, 12 (44.4%) were Mf-positive, and of the 28 slides examined from Ili’ili/Vaitogi/Futiga, 9 (32.1%) were Mf-positive. Amongst the 32 Ag-positive people from Fagali’i, 31 (96.9%) were positive for Bm14 and Wb123 antibodies, and all were positive for Bm33 antibody. Amongst the Ag-positive people from Ili’ili/Vaitogi/Futiga, 23 (63.9%) were positive for Bm14, 30 (83.3%) for Wb123, and 34 (94.4%) for Bm33 antibodies. Fig 6 shows the seroprevalence of Ag and antibodies by age groups for residents in the two hotspots, compared to randomly selected villages.

TAS-3 also included 139 children from the two hotspots, including seven from Fagali’i, and 132 from Ili’ili/Vaitogi/Futiga. Of the nine Ag-positive children identified in TAS-3, two were residents of Fagali’i and one was a resident of Ili’ili/Vaitogi/Futiga. Adjusted Ag prevalence of TAS-3 children in these two hotspots were 18.8% (95% CI 4.3–54.7%) and 0.9% (95% CI 0.1–6.0%), respectively.

#### Household and community members of Ag-positive children from TAS-3

The nine index children identified in TAS-3 lived in nine households in six different villages. A total of 58 household members were surveyed from the nine households. Of these, 32 (55.2%) were female, and 52 (89.7%) were aged ≥8 years, representing 80% of the reported 65 household members aged ≥8 years who lived in these households. Of the 58 household members tested, 12 (20.7%, 95% CI 11.2–33.4%) were Ag-positive; Ag prevalence was 0% in children aged <8 years (n = 6), 27.8% (95% CI 9.7–53.5%) in those aged 8–17 years (n = 18), and 20.6% (95% CI 8.7–37.9%) in those aged ≥18 years (n = 34). Microfilaria slides were available for 9 of the 12 Ag-positive household members, and two of these (22.2%) were Mf-positive. Amongst Ag-positive household members, 83.3% (95% CI 51.6–97.9%) were positive for Bm14, 75.0% (95% CI 42.8–94.5%) for Wb123, and 91.7% (61.5–99.8%) for Bm33 antibodies.

**Fig 7:**
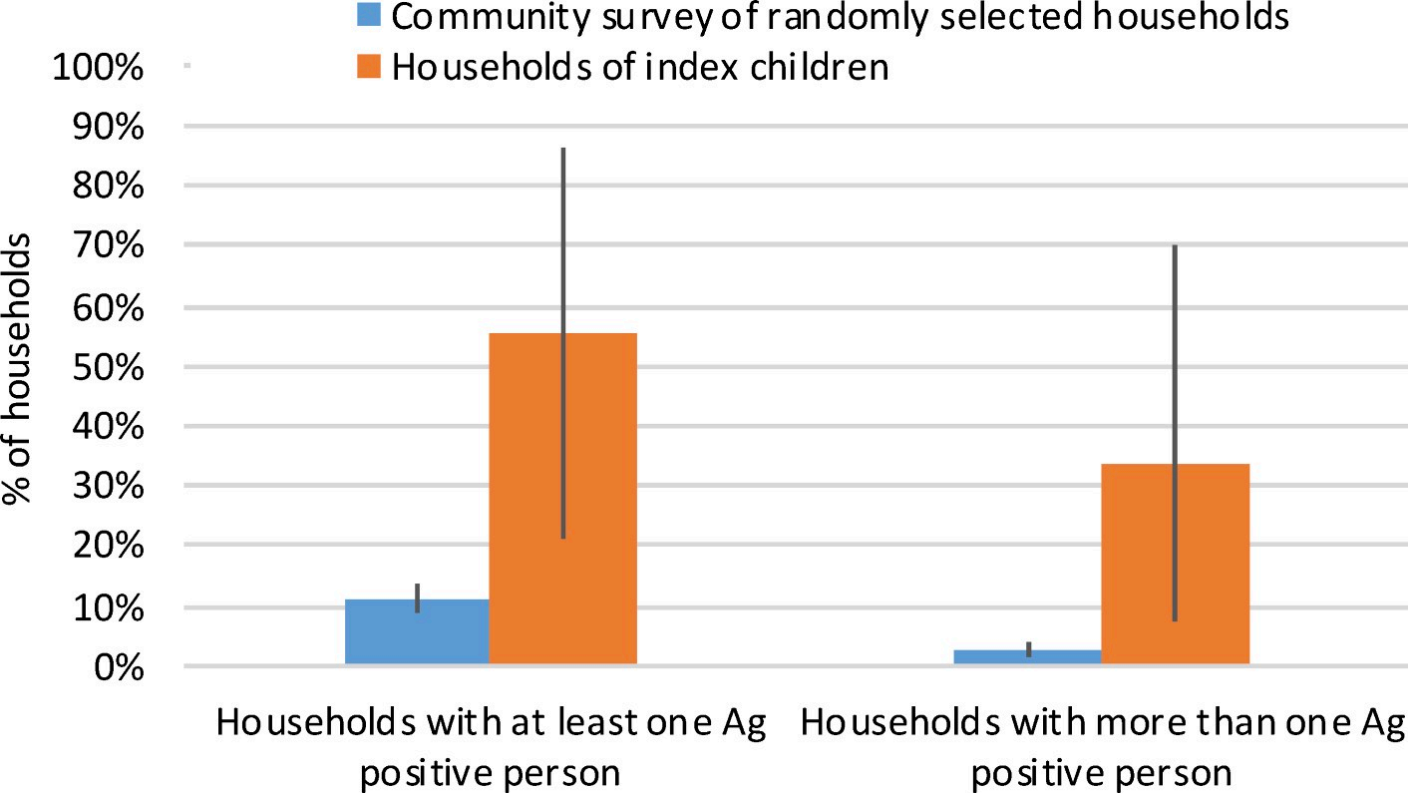
Percentage of households of index children versus randomly selected households in community survey where i) at least one antigen-positive person was identified, and ii) more than one antigen-positive person was identified.

At least one Ag-positive individual (in addition to the index child) was identified from five (55.6%) of the nine index households, and more than one additional Ag-positive person (range 2–5) was identified in three (33.3%) of these households. In comparison, the 2016 community survey of randomly selected villages identified at least one Ag-positive person from 79 (11.1%) of the 711 households, and more than one Ag-positive person (range 2–4) in only 18 (2.5%) of the households (Fig 7).

**Fig 8:**
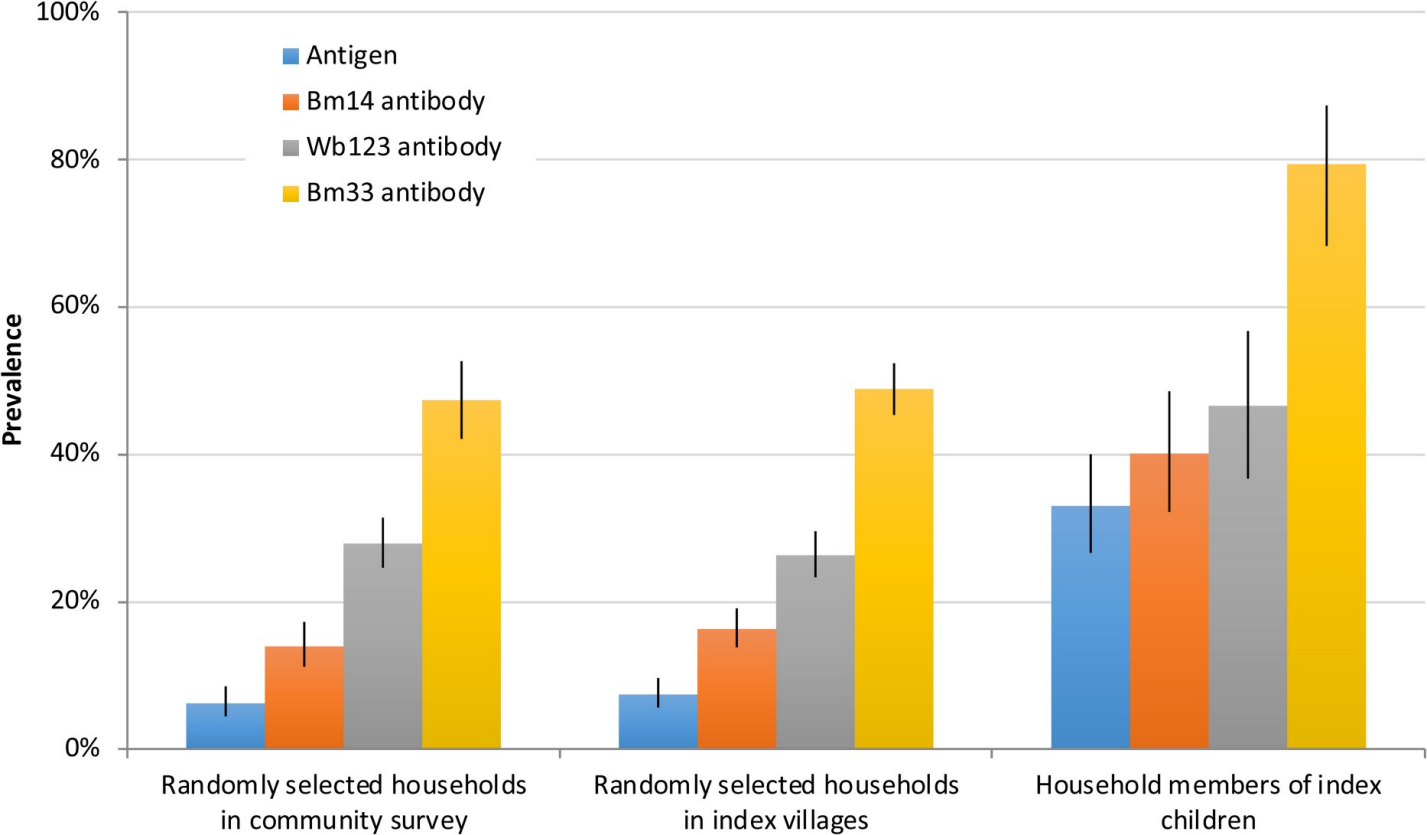
Adjusted seroprevalence of Ag and antibodies in i) randomly selected households in randomly selected villages in community survey (n = 2496 for Ag, 2498 for antibodies), ii) randomly selected households in index villages (n = 924 for Ag, 922 for antibodies), and iii) household members of index children (n = 52 for Ag, 50 for antibodies). To enable direct comparisons, results for all groups were limited to participants aged ≥8 years.

Of the six index villages, four (Vaitogi, Pago Pago, Fagatogo, and Tafuna) were part of the randomly selected villages for the community survey in 2016. One of these (Vaitogi) was also part of a previously identified transmission hotspot [3,10] and the fifth village (Fagali’i) was purposefully included because previous studies identified it as a transmission hotspot [3,4]. The sixth village (Faga’alu) was not surveyed in 2016, so no village-level seroprevalence data were available. From the five index villages, results for Ag and antibodies in those aged ≥8 years were available for 924 and 922 persons, respectively. Fig 8 shows that seroprevalence of Ag and antibodies were significantly higher in index households (based on 5 of the 6 villages where community-level data were available) compared to randomly selected households in index villages or in randomly selected villages.

### Risk factors analysis

For the community survey of residents aged ≥8 years, the prevalence of demographic and behavioural factors, and their association with positive seromarkers and Mf are summarised in Tables 1 (for Ag and Mf) and 2 (for antibodies). For all seromarkers and Mf, both univariable and multivariable logistic regression showed that males, older age groups, and residents of Fagali’i were significantly more likely to be positive. While indoor workers were less likely to be positive, those who worked outdoors, at the tuna cannery and in other places were significantly positively associated with all seromarkers on univariable and multivariable analyses. Outdoor workers were more likely to be Mf-positive on univariable (but not multivariable) analyses, and other workers were more likely to be Mf-positive in both univariable and multivariable analyses. Those who had travelled to an LF-endemic area in the past 12 months (96.2% to Samoa) were more likely to be seropositive for Bm33 antibody (but not the other seromarkers or Mf) on both univariable and multivariable regression. The minority (15.8%) who had travelled to non-LF endemic areas in the past 12 months (most commonly to mainland USA, Hawaii, New Zealand, and Australia) were less likely to be seropositive for Ag and antibodies; this result may be confounded by socioeconomic status rather than any causal link. The number of years lived in American Samoa was not associated with Ag or antibody seropositivity, but those who had lived in American Samoa for over 10 years were more likely to be Mf-positive on univariable analysis. Participants who reported taking MDA in the past were more likely to be seropositive for Ag and antibodies on univariable analyses, but these were not significant on multivariable analyses (Tables 1 and 2).

**Table 1:** Community survey of residents aged ≥8 years: Summary of demographic and behavioural factors, and their associations with antigen-positive and microfilaria-positive results on univariable and multivariable logistic regression.

	Participants	Antigen positive (by FTS)					Microfilaria positive				
			Univariable		Multivariable			Univariable		Multivariable	
	N (%)	N (%)	uOR	p	aOR	p	N (%)	uOR	p	aOR	p
**Total samples**	2698 (100)	2685					2663				
**Total positive**		137 (5.1)					34 (1.3)				
**Gender**											
Female Male	1469 (54.5) 1229 (45.6)	38 (2.6) 99 (8.1)	Ref 3.33	**<0.001**	Ref 3.01	**<0.001**	10 (0.7) 24 (2.0)	Ref 2.97	**0.004**	Ref 2.42	**0.028**
**Age groups**											
8 to 12 years 13 to 17 years ≥ 18 years	418 (15.5) 387 (14.3) 1893 (70.2)	9 (2.2) 6 (1.6) 122 (6.5)	Ref 0.72 3.15	0.529 **0.001**	Ref 0.76 2.18	0.605 **0.047**	1 (0.24) 1 (0.26) 32 (1.7)	Ref 1.08 7.26	0.959 0.051		
**Live in known hotspot**											
No Ili’ili/Vaitogi/Futiga Fagali’i	1987 (73.6) 624 (23.1) 87 (3.2)	69 (3.5) 36 (5.8) 32 (37.2)	Ref 1.69 16.38	**0.012** **<0.001**	Ref 1.79 15.81	**0.007** **<0.001**	13 (0.7) 9 (1.5) 12 (14.8)	Ref 2.23 26.14	0.066 **<0.001**	Ref 2.30 23.14	0.06 **<0.001**
**Work location**											
Indoor Outdoor Tuna cannery Other	1818 (67.4) 57 (2.1) 133 (4.9) 690 (25.6)	50 (2.8) 12 (21.4) 13 (9.9) 62 (9.1)	Ref 9.63 3.86 3.53	**<0.001** **<0.001** **<0.001**	Ref 2.61 2.50 2.30	**0.025** **0.010** **<0.001**	9 (0.5) 3 (5.6) 1 (0.8) 21 (3.1)	Ref 11.75 1.55 6.44	**<0.001** 0.679 **<0.001**	Ref 3.21 1.70 5.59	0.123 0.620 **<0.001**
**Time lived in AS**											
<5 years 5 to 10 years >10 years	494 (18.3) 186 (6.9) 2018 (74.8)	27 (5.5) 7 (3.8) 103 (5.1)	Ref 0.67 0.93	0.359 0.741			2 (0.4) 1 (0.5) 31 (1.6)	Ref 1.32 3.81	0.821 0.067 **<0.001**		
**Ever taken MDA**											
No Yes Unknown	1518 (57.7) 1115 (42.4) 65 (2.4)	60 (4.0) 72 (6.5)	Ref 1.69	**0.004**			13 (0.9) 19 (1.4)	Ref 2.03	0.050		
**Travel to LF endemic area in past 12 months**											
No Yes Unknown	2250 (83.5) 446 (16.5) 2 (0.1)	113 (5.1) 24 (5.4)	Ref 1.08	0.754			29 (1.3) 5 (1.1)	Ref 0.87	0.768		
**Travel to non-LF endemic areas in past 12 months**											
No Yes Unknown	2271 (84.2) 425 (15.8) 2 (0.1)	126 (5.6) 11 (2.6)	Ref 0.45	**0.013**	Ref 0.41	**0.008**	32 (1.4) 2 (0.5)	Ref 0.33	0.129		

uOR = unadjusted odds ratio, aOR = adjusted odds ratio, AS = American Samoa

**Table 2:** Community survey of residents aged ≥8 years: Summary of demographic and behavioural factors, and their associations with positive Bm14, Wb123, and Bm33 antibodies on univariable and multivariable logistic regression.

	Participants	Bm14 antibody positive					Wb123 antibody positive					Bm33 antibody positive				
			Univariable		Multivariable			Univariable		Multivariable			Univariable		Multivariable	
	N (%)	N (%)	uOR	p	aOR	p	N (%)	uOR	P	aOR	p	N (%)	uOR	P	aOR	p
**Total samples**	2689 (100)	2689					2689					2689				
**Total positive**		356 (13.2)					697 (25.9)					1229 (45.7)				
**Gender**																
Female Male	1469 (54.5) 1229 (45.6)	137 (9.36) 219 (17.9)	Ref 2.10	**<0.001**	Ref 2.02	**<0.001**	293 (20.0) 404 (33.0)	Ref 1.96	**<0.001**	Ref 1.99	**<0.001**	617 (42.2) 612 (49.9)	Ref 1.37	**<0.001**	Ref 1.36	**<0.001**
**Age groups**																
8 to 12 years 13 to 17 years ≥ 18 years	418 (15.5) 387 (14.3) 1893 (70.2)	19 (4.6) 31 (8.1) 306 (16.2)	Ref 1.84 4.07	**0.043** **<0.001**	Ref 2.05 3.43	**0.023** **<0.001**	49 (11.7) 74 (19.2) 574 (30.4)	Ref 1.79 3.29	**0.003** **<0.001**	Ref 1.96 3.35	**0.001** **<0.001**	124 (29.7) 135 (35.1) 970 (51.4)	Ref 1.28 2.51	0.102 **<0.001**	Ref 1.34 2.23	0.064 **<0.001**
**Live in known hotspot**																
No Ili’ili/Vaitogi/Futiga Fagali’i	1987 (73.6) 624 (23.1) 87 (3.2)	216 (10.9) 78 (12.5) 62 (71.3)	Ref 1.17 20.25	0.263 **<0.001**	Ref 1.22 22.38	0.165 **<0.001**	449 (22.7) 177 (28.5) 71 (81.6)	Ref 1.36 15.13	**0.003** **<0.001**	Ref 1.43 15.99	**0.001** **<0.001**	805 (40.7) 341 (54.8) 83 (95.4)	Ref 1.77 30.29	**<0.001** **<0.001**	Ref 1.85 30.20	**<0.001** **<0.001**
**Work location**																
Indoor Outdoor Tuna cannery Other	1818 (67.4) 57 (2.1) 133 (4.9) 690 (25.6)	159 (8.8) 20 (35.1) 29 (22.0) 148 (21.5)	Ref 5.61 2.92 2.84	**<0.001** **<0.001** **<0.001**	Ref 2.09 1.92 2.03	**0.033** **<0.001** **<0.001**	375 (20.7) 31 (54.4) 52 (39.4) 239 (34.6)	Ref 4.56 2.49 2.03	**<0.001** **<0.001** **<0.001**	Ref 1.82 1.53 1.40	**0.047** **0.034** **0.003**	719 (39.7) 37 (64.9) 78 (59.1) 395 (57.3)	Ref 2.81 2.19 2.03	**<0.001** **<0.001** **<0.001**	Ref 1.41 1.61 1.53	0.258 **0.014** **<0.001**
**Time lived in AS**																
<5 years 5 to 10 years >10 years	494 (18.3) 186 (6.9) 2018 (74.8)	66 (13.4) 24 (13.0) 266 (13.2)	Ref 0.96 0.98	0.880 0.909			135 (27.4) 46 (24.9) 516 (25.7)	Ref 0.88 0.91	0.500 0.416			231 (47.0) 95 (51.4) 903 (44.9)	Ref 1.19 0.92	0.307 0.408		
**Ever taken MDA**												646 (42.7)				
No Yes Unknown	1518 (57.7) 1115 (42.4) 65 (2.4)	165 (10.9) 184 (16.6)	Ref 1.62	**<0.001**			335 (22.1) 346 (31.2)	Ref 1.59	**<0.001**			554 (49.9) 1200 (45.7)	Ref 1.34	**<0.001**		
**Travel to LF endemic area in past 12 months**																
No Yes Unknown	2250 (83.5) 446 (16.5) 2 (0.1)	290 (12.9) 66 (14.9)	Ref 1.18	0.272			568 (25.3) 128 (28.8)	Ref 1.19	0.124			997 (44.5) 231 (52.0)	Ref 1.36	**0.003**	Ref 1.28	**0.025**
**Travel to non-LF endemic areas in past 12 months**																
No Yes Unknown	2271 (84.2) 425 (15.8) 2 (0.1)	320 (14.2) 36 (8.5)	Ref 0.56	**0.002**	Ref 0.47	**<0.001**	626 (27.7) 71 (16.7)	Ref 0.52	**<0.001**	Ref 0.44	**<0.001**	1055 (46.6) 174 (40.9)	Ref 0.79	**0.031**	Ref 0.67	**0.001**

uOR = unadjusted odds ratio, aOR = adjusted odds ratio, AS = American Samoa

### Intra-cluster correlation (ICC)

**Fig 9:**
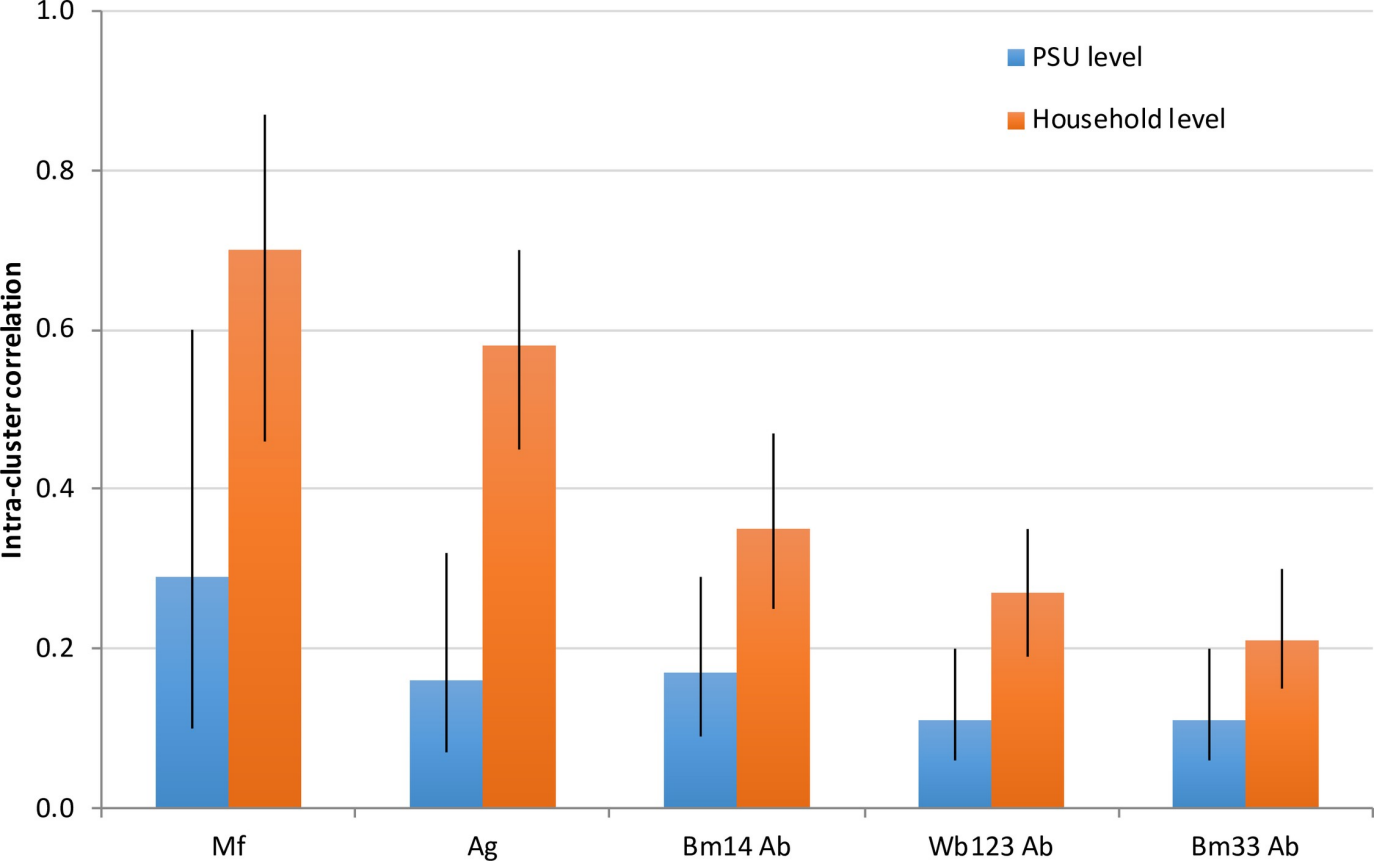
Intra-cluster correlation (ICC) and 95% CI at PSU and household levels for positive results for microfilaria, Ag, and antibodies (Bm14, Wb123, and Bm33).

ICCs were higher at the household than PSU levels for Mf and all seromarkers, indicating that clustering is more intense at the household level. ICCs were highest for Mf, followed by Ag, Bm14 Ab, Wb123 Ab, and Bm33 Ab (Fig 9).

### D. Number needed to test (NNTest)

**Fig 10:**
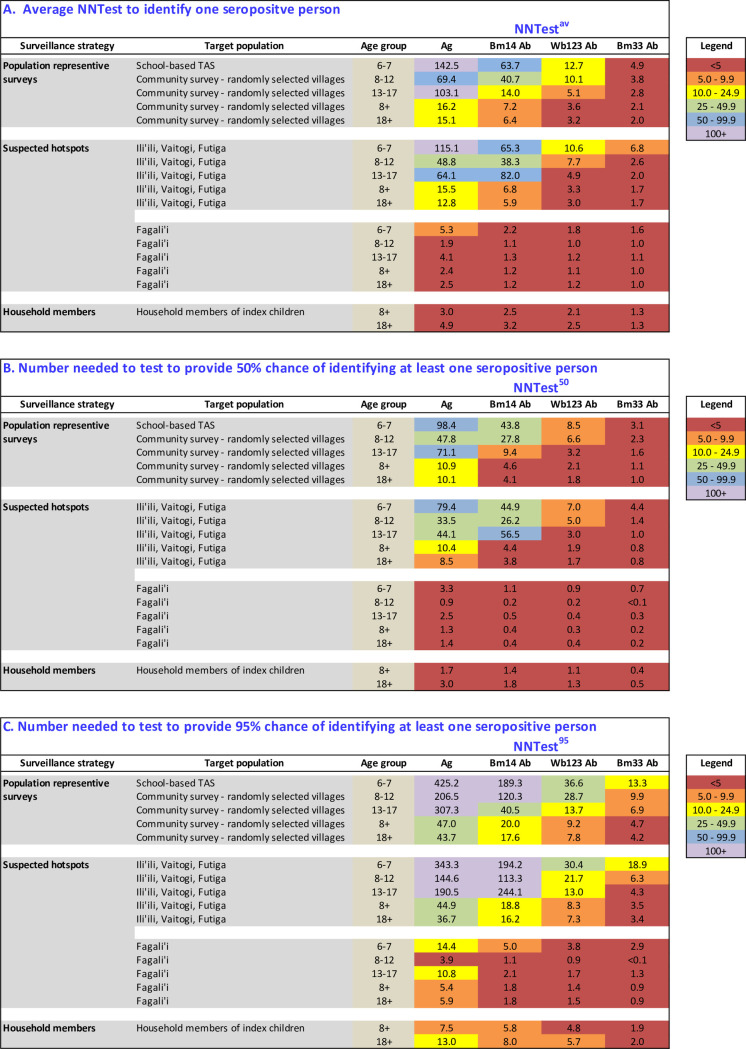
Number need to test for different surveillance strategies, target populations, and age groups using antigen and Bm14, Wb123, and Bm33 antibodies. A) Average number needed to test to identify one seropositive person (NNTest^av^), B) Number needed to test to provide a 50% chance of identifying at least one seropositive person (NNTest^50^), and C) Number needed to test to provide a 95% chance of identifying at least one seropositive person (NNTest^95^).

NNTest^av^, NNTest^50^, and NNTest^95^ were calculated for subgroups based on age groups and survey strategy: TAS, population representative surveys of older age groups, and targeted surveillance of high risk groups (hotspots, household members of Ag-positive children) (Fig 10). For all subgroups (except for 13–17 year olds in IVF), all three NNTest measures were highest for Ag, followed by Bm14, Wb123, and Bm33 antibodies. NNTest also generally decreased as the age of the target population increased. Population representative surveys such as TAS and community surveys were associated with significantly higher NNTest than more targeted surveillance strategies.

If targeted surveillance was conducted in households of index children, the NNTest^av^ was ≤3 regardless of the diagnostic test used. In these households, where additional Ag-positive individuals were identified from five of nine households, an average of only 1.8 households needed to be surveyed in order to find at least one Ag-positive person, compared to an average of 9.0 households (5 times as many) for randomly selected households.

**Fig 11:**
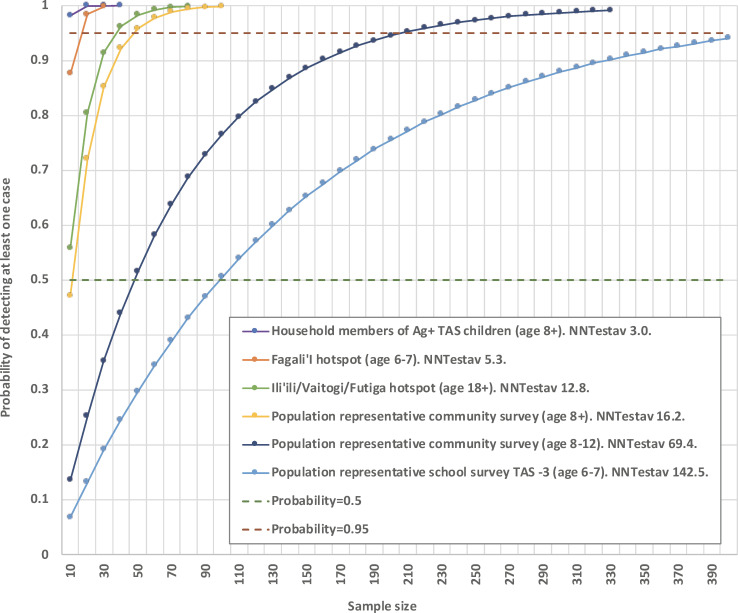
Probability of detecting at least one Ag-positive case based on sample size and selected survey strategies with different levels of NNTest^av^ (assumes that Ag-positive cases are independent events). Strategies shown include population representative surveys of 6–7 year-olds, 8–12 year-olds, and those ≥8 years old; hotspot villages; and household members of Ag-positive children identified in TAS (index children).

Fig 11 shows the probability of detecting at least one Ag-positive case based on selected survey strategies with different levels of NNTest^av^ and sample size (assuming that positive cases are independent events). For example, for TAS-3 of 6-7-year-old children (NNTest^av^ = 142.5), over 400 children need to be tested to provide >95% probability of detecting at least one Ag-positive case if they exist. In contrast, for household members of Ag-positive children identified in TAS-3 (NNTest^av^ = 3.0), less than 10 people need to be tested to provide the same confidence. S1 Fig shows the probability of detecting at least one positive case based on sample size and NNTest^av^ of 5, 10, 20, 50, 100, and 150 (assuming that positive cases are independent events).

## Discussion

For LF elimination programmes, the goals of surveillance are to identify any remaining Ag-positive people and transmission hotspots, and pick up signals of resurgence at an early stage. Our study demonstrated some potential strategies for strengthening surveillance and improving the likelihood of achieving these goals. In the post-MDA setting, the ability of surveillance strategies to efficiently identify signals of residual transmission and/or resurgence is critical but challenging, particularly if infected persons are not randomly distributed. Our study quantified the degree to which strategies that specifically target high-risk populations and locations can improve sampling efficiency at identifying such signals, and provided evidence to suggest that these strategies should be considered together with population representative surveys such as TAS. Compared to current recommendations for TAS in 6–7 year-old children, we found that the NNTest was significantly lower if testing older children and/or adults, conducting targeted surveillance (of known hotspots and households of index children), using antibodies, or a combination of these strategies.

It is important to note that although the sampling strategies with lower than NNTest^av^ might be more efficient for identifying infected persons, the samples are not population representative and cannot be used to provide accurate estimates of population-level seroprevalence. Therefore, population representative surveys such as TAS will be required to estimate true prevalence as well as provide a starting point to inform the targeted strategies. However, strategies with lower NNTest^av^ are more likely to identify rare events (if they exist) such as Ag-positive people in the post-MDA setting. For post-MDA surveillance, identifying any remaining Ag-positive people (which might provide indications of hotspots and/or resurgence) is arguably strategically more important than having accurate population-level estimates of infection prevalence. In American Samoa, considering that LF prevalence is disproportionately higher in some groups (e.g. males, older individuals) [3] and transmission has been shown to be highly focal in the post-MDA setting [3–5,7,8], targeted surveillance of high-risk persons and locations could significantly reduce the NNTest^av^, thereby improving the precision and cost-effectiveness of post-MDA surveillance.

Higher seroprevalence of Ag and antibodies in older age groups is a common finding in LF surveys across the world. The current TAS recommendation for testing 6–7 year-old children is based on the rationale that detection of infections in young children (who were born after MDA had started) provides evidence of ongoing transmission. Also, conducting school-based surveys of children is generally logistically simpler and cheaper than community-based household surveys [5], and a large population can be tested at relatively low cost. Furthermore, TAS is already a WHO-approved tool that country programs are familiar with and have the resources to implement. However, the low prevalence of infection in young children (6–7 years) means that the NNTest is significantly higher compared to testing older age groups (e.g. school-based surveys of children aged 8–12 years or 13–17 years), resulting in reduced efficiency and cost-effectiveness of surveys. For a manageable sample size, there is a higher chance of missing early signals of resurgence in 6–7 year-olds than in older persons Our study showed that population representative surveys of older children have the potential to reduce NNTest, and cost-effectiveness could be maintained by conducting the surveys at schools if school attendance is high, e.g. testing age 8–12 year-olds in elementary schools, or age 13–17 year-olds at secondary school. Furthermore, testing 8–12 year-olds in TAS-2, TAS-3, and beyond is consistent with the rationale behind TAS, i.e targeting age groups who have lived their entire lives during and/or after MDA, so any positive cases would indicate incident infection and therefore ongoing transmission. Population representative surveys in communities have the potential to further reduce NNTest (because adults can be included) and provide more detailed epidemiological information than school-based surveys, but are logistically more complex and more expensive [16]. While positive Ag in children represent more recent infections, active infections in adults should also be considered as important signals of ongoing transmission.

Our findings provide strong evidence to support targeted surveillance and/or presumptive treatment of household members of infected persons. Household members of index children had significantly higher seroprevalence than any other groups. Higher seroprevalence in household members compared to community members of index children, together with higher ICCs for all seromarkers at household than PSU level, indicate that infections are intensely clustered at the household level. Based on current TAS guidelines, household members of Ag-positive children identified through TAS should be tested and/or treated, but this is not always done. Furthermore, there are no evidence-based recommendations for using information about Ag-positive children from the TAS to inform further surveillance activities. Considering that young children spend most of their time at home or at school, it is plausible that index children could be used as sentinels for more targeted and therefore more cost-effective surveillance. In our study, NNtest^av^ was <5 for household members of index children regardless of the seromarkers used, and more than half the index households had at least one Ag-positive person. Testing/treating household members would therefore be a very cost effective public health strategy for reducing reservoirs of infection, and also clinically important for reducing the risk of long-term morbidity in individuals. If future surveillance recommendations include other strategies (e.g. testing older children at school, opportunistic testing of adult workers [4,22]), testing/treating all household members of seropositive persons should also be considered, regardless of how they were identified. Population representative survey strategies that reduce NNTest can further improve the efficiency of identifying sentinels that could lead us to more households with high prevalence of infection. It should be noted that there is currently no clear evidence that a single treatment dose is sufficient to permanently clear infection, but the use of the triple-drug combination of ivermectin, DEC, and albendazole (IDA) is more likely to be effective than 2-drug combinations [30]. Further studies are required to determine whether a single treatment with IDA is sufficient, and if not, the number of treatments required for long-term clearance.

Targeted surveillance of known hotspots is another potential strategy for reducing NNTest. In American Samoa, hotspots were identified by previous studies conducted in 2010 and 2014. In 2016, some of these areas again had higher seroprevalence of Ag and antibodies, suggesting ongoing transmission. While we recognise that there are many potential definitions of a hotspot and the term is used in many different contexts, we have chosen a relative definition in this study to determine whether more targeted interventions should be considered in these areas, regardless of the overall prevalence in the evaluation unit. In other contexts, hotspots are sometimes defined using only absolute terms, e.g. Ag prevalence above WHO recommended thresholds. Currently, there is limited information on the geographic size of transmission hotspots, and whether this differs between places, environmental conditions, vector species, time since MDA, or other factors. Our previous study in American Samoa suggested cluster size of 1.2 to 1.5km [3], while a study in Haiti found a significantly higher likelihood of being Ag-positive if living within 20 metres of index cases [11]. A study in India defined transmission hotspots as locations with population of >250 people with Ag prevalence of ≥2%, and found 17 hotspots with population size ranging from 252 to 2318 (mean 800) [31]. Considering the focal nature of residual hotspots, geographically driven surveillance strategies (e.g. surveillance of near neighbours) may improve precision and reduce cost. To optimise such strategies, further studies are required to determine temporal and spatial characteristics of hotspots, factors that influence cluster size, risk of infection in near neighbours, whether targeted surveillance of these neighbours is warranted, and how large the surveillance buffer should be. In the future, geostatistical and other spatially-explicit models may be able to predict hotspots based on known locations of infections, environmental factors, and social connectivity [32–35].

Our study found that outdoor workers were more likely to be seropositive for Ag and antibodies. We did not include work location in our NNTest matrices, but it is possible to include this and other risk factors (e.g. male gender, molecular xenomonitoring data) when planning surveillance, especially if it is feasible to develop targeted surveillance strategies for these subpopulations or locations.

Currently, Ag is the key seromarker used for LF surveillance. Considering that antibody prevalence is typically higher than Ag prevalence, using antibodies for surveillance (possibly together with Ag) could reduce NNTest. Studies in children in Samoa and Sri Lanka found that antibody was more sensitive than Ag for detecting residual transmission of LF in the post-MDA setting [36,37]. The two main barriers to using antibodies are poor understanding of the changes in antibody patterns over time, and the lack of a rapid diagnostic test that can be used in field surveys. Antigen detected by tests such as FTS [38,39] is produced by adult filarial worms in the lymphatic system and circulates in the peripheral blood during the worm’s lifespan and for an unknown period thereafter. The Wb123 antibody was identified from a library generated from L3 larval stages of *W*. *bancrofti* [40], and is detectable in the early stages of infection, but also persists for long periods during and after infection in adults [41]. Model-based simulations estimate that L3 antibodies may persist for about 9.6 years [42]. The Bm14 antibody was identified from a cDNA library screened using sera from microfilariaemic people [43,44], and may persist for many years post-infection. Bm33 antibody was also identified in a *B*.*malayi* cDNA library as a major cross-reacting immunogen in *W*. *bancrofti* [45]. The duration of persistence of Bm14 and Bm33 antibodies is long (many years) but exact duration is not well known. The discordance between Ag and antibodies results in young children indicate that serological patterns in recently acquired infections are complex, and each seromarker most likely provides different information about infection and immune status. Further studies are needed to understand how serological patterns of Ag and antibodies evolve over time, the sensitivity and specificity of antibodies as indicators of recent or active infection, and their utility as surveillance tools.

The NNTest results presented in this paper reflect the epidemiology of LF in American Samoa. For other locations, NNTest might differ depending on infection prevalence, the degree of community and household level clustering (ICCs), and the characteristics of hotspots. For future cluster surveys, sample size calculations should adjust for design effect at both the PSU and household levels. Considering that ICC is higher for Ag than antibodies, the design effect will be also higher. For strategies with already high NNTest, the need to multiply this by a large design effect could significantly further increase the sample size and the primary and/or secondary sampling units required.

Other strategies that could improve the cost-effectiveness of post-MDA surveillance include integrating surveillance with other diseases or public health programs, opportunistic screening of large populations (such as workers, military recruits and blood donors), and molecular xenomonitoring of mosquitoes [4,16,46]. Our previous work in American Samoa demonstrated that community-based survey of older age groups may provide earlier, more sensitive, and/or more geographically precise signals of ongoing transmission than TAS [5], but was logistically more challenging and more expensive. Screening of adults could therefore reduce NNTest [46], and if conducted through an efficient sampling strategy (e.g. opportunistic screening at workplaces or clinics [4]), it is potentially possible to achieve the desirable combination of low NNTest and low cost. In American Samoa [16], Sri Lanka [8], and India [47], molecular xenomonitoring of mosquitoes has also been shown to potentially provide earlier and more sensitive signals of transmission than TAS, and could be useful for informing more targeted surveillance. However, molecular xenomonitoring requires access to entomological expertise for mosquito identification and a laboratory for molecular testing, which may be beyond the reach of many developing countries.

**Fig 12:**
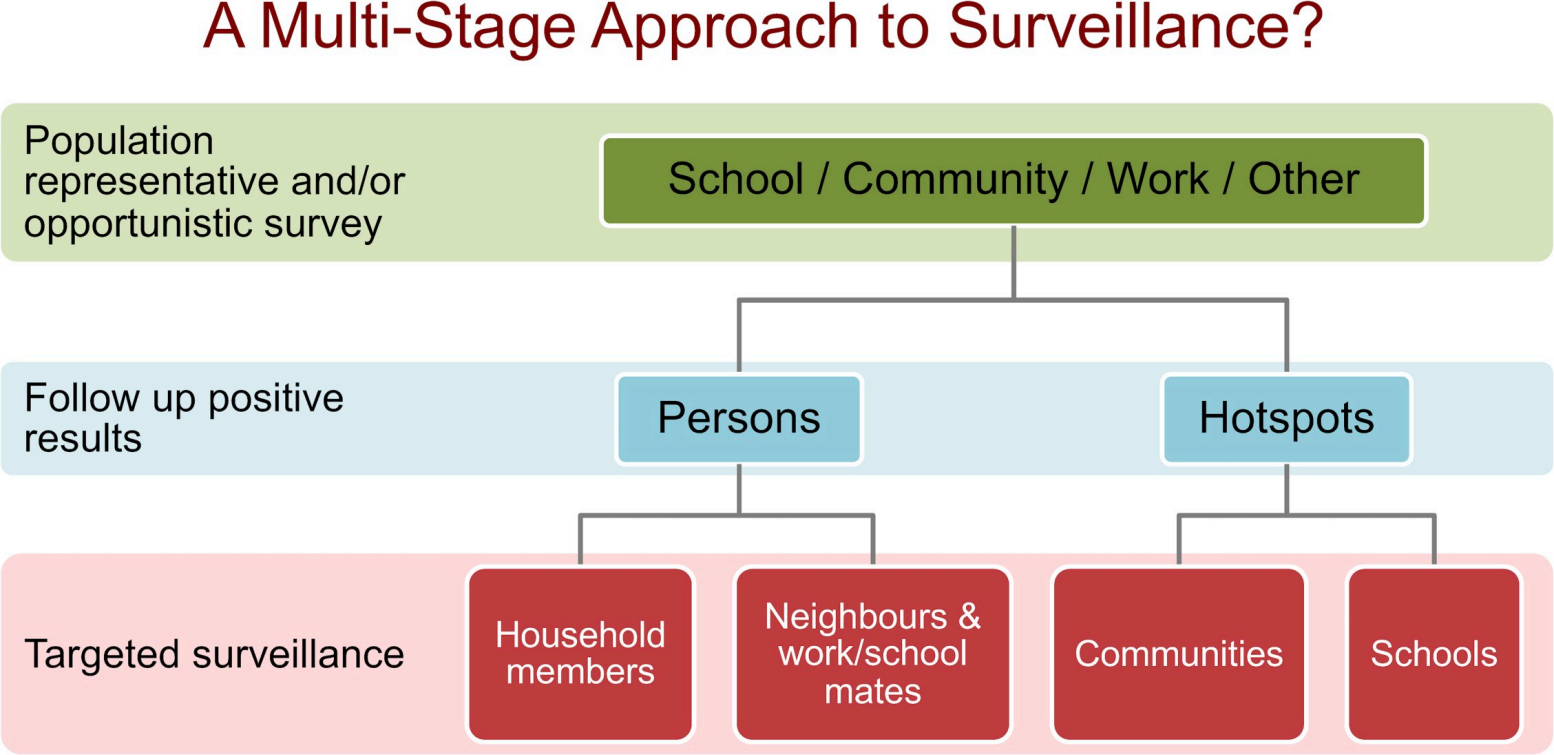
Potential framework for a multi-stage surveillance strategy for LF elimination programs.

Taking together findings from our current and previous studies, an approach for improving effectiveness and efficiency of post-MDA surveillance could be a multi-stage test-and-treat strategy for identifying and eliminating residual foci of transmission. A multi-stage operational framework (Fig 12) could start with population representative sampling that takes into account the NNTest for different target age groups, and operational costs and logistics for different survey sites (e.g. community, school, work sites). Opportunistic screening, such as at work sites, could also be considered as a first step. The next step would be a more intensive targeted sampling of any high-risk groups (including household members of infected persons) and high prevalence locations and hotspots. Targeted sampling should consider all relevant data from the population representative survey and make the most use of any clues about the demographics and locations of high-risk persons. For example, a multi-stage strategy could start with a school-based survey of older children, followed by active targeted surveillance or ‘contact tracing’ of index households, and more intensive surveillance of hotspots. For both the population representative and targeted sampling stages, programs need to decide on the level of uncertainty that they are prepared to accept, and balance this against the sample size (and cost) required. In populations with low NNTest (e.g. in hotspots), a small increase in sample size is associated with a steep increase in the probability of identifying at least one more case; targeted sampling in these areas would therefore be highly likely to identify other infected persons and provide further sentinels. In contrast, in populations with very high NNTest (e.g. TAS of 6–7 year-olds), a relatively large increase in sample size is required to increase the probability of detecting one case, particularly as the desired probability approaches one (Figs 11 and S1). Molecular xenomonitoring and other strategies could potentially be included in the multi-stage approach once there is sufficient evidence about their effectiveness, including the interpretation of the results and the actions required.

The strengths of our study include high-resolution data that enabled linkage of individuals by households and communities, testing and surveying household members of index children, and knowledge of previously identified hotspots which we could further assess. By conducting a school-based TAS and a community survey in parallel, we were also able to accurately compare results between the two survey methods.

Our results should be considered in light of limitations. The study was designed to detect differences between 6–7 year-olds and those aged ≥8 years, but not the smaller age bands of 8–12 years, 13–17 years, and ≥18 years. Despite this, significant difference in seroprevalence between age groups allowed detailed comparisons. Similarly, the study was not powered to detect differences between PSUs, but significantly higher seroprevalence were noted in the previously identified hotspots. The sample size in some subpopulations were very small, resulting in wide 95% CI for prevalence estimates. For example, Ag prevalence estimates for 6–7 year-old children in Fagali’i were based on two positives out of nine children tested (adjusted Ag prevalence 18.8%, 95% CI 4.3–54.7%). S1 Fig can be used to determine the relationship between different NNTest and the probability of detecting at least one case using different sample sizes. The study was conducted in American Samoa, a remote tropical island location where LF is caused by *Wuchereria bancrofti* and transmitted by *Aedes* mosquitoes; the concepts explored in our study could be applied to other locations, but the quantitative results might differ.

Another limitation of surveillance strategies that rely on population-representative surveys (e.g. cluster sampling) is that they may miss residual infections and hotspots altogether. Therefore, the overall success of any targeted surveillance program and/or test-and-treat program depends on the sensitivity of the initial survey(s). Although TAS in American Samoa included all elementary schools, this is not the case for most countries, where only a small proportion of schools or communities are tested. Improving the representativeness and sensitivity of the initial survey is therefore crucial for overall success, e.g. geospatial models to guide adaptive sampling of clusters, and more strategic sampling in TAS-2 and TAS-3 to ensure greater representativeness of the entire evaluation unit. Currently, there is limited evidence on the Ag or antibody thresholds for conducting targeted MDA at a broader population level; this information is important for ensuring that undetected infections are also treated.

## Conclusion

Historical evidence demonstrates that LF can resurge in the Pacific islands even when infection rates have dropped to very low levels [48,49]. Possible reasons include the combination of efficient vectors, highly mobile population [33], outdoor lifestyle, MDA coverage/participation rates [21], and remote/isolated populations that make drug distribution and surveillance a challenge. Public health resources are usually limited and need to be shared amongst many health priorities. Taking these factors together, long-term success of the LF elimination programme in the Pacific Islands (and most probably elsewhere) will require highly effective and efficient surveillance strategies that maximise the likelihood of identifying residual foci of transmission and detecting early signals of any resurgence.

## Supporting information

S1 Checklist STROBE checklist for cross-sectional studies. (DOCX)

S1 Fig Probability of detecting at least one positive case based on sample size and NNTest^av^ of 5, 10, 20, 50, 100, and 150 (assumes that positive cases are independent events). (TIFF)

S1 Table Probability of selection and post-stratification weights of different subgroups. (DOCX)

S2 Table Adjustments used for different subgroups. (DOCX)

S1 Text Methods used to calculate seroprevalence adjusted for age, sex, and survey design. (DOCX)
