# Supplementary figures and images for: Potential strategies for strengthening surveillance of lymphatic filariasis in American Samoa after mass drug administration: Reducing ‘number needed to test’ by targeting older age groups, hotspots, and household members of infected persons
Source: PLoS Negl Trop Dis. 2020 Dec 28;14(12):e0008916. doi: 10.1371/journal.pntd.0008916 (PMC7872281; doi:10.1371/journal.pntd.0008916)

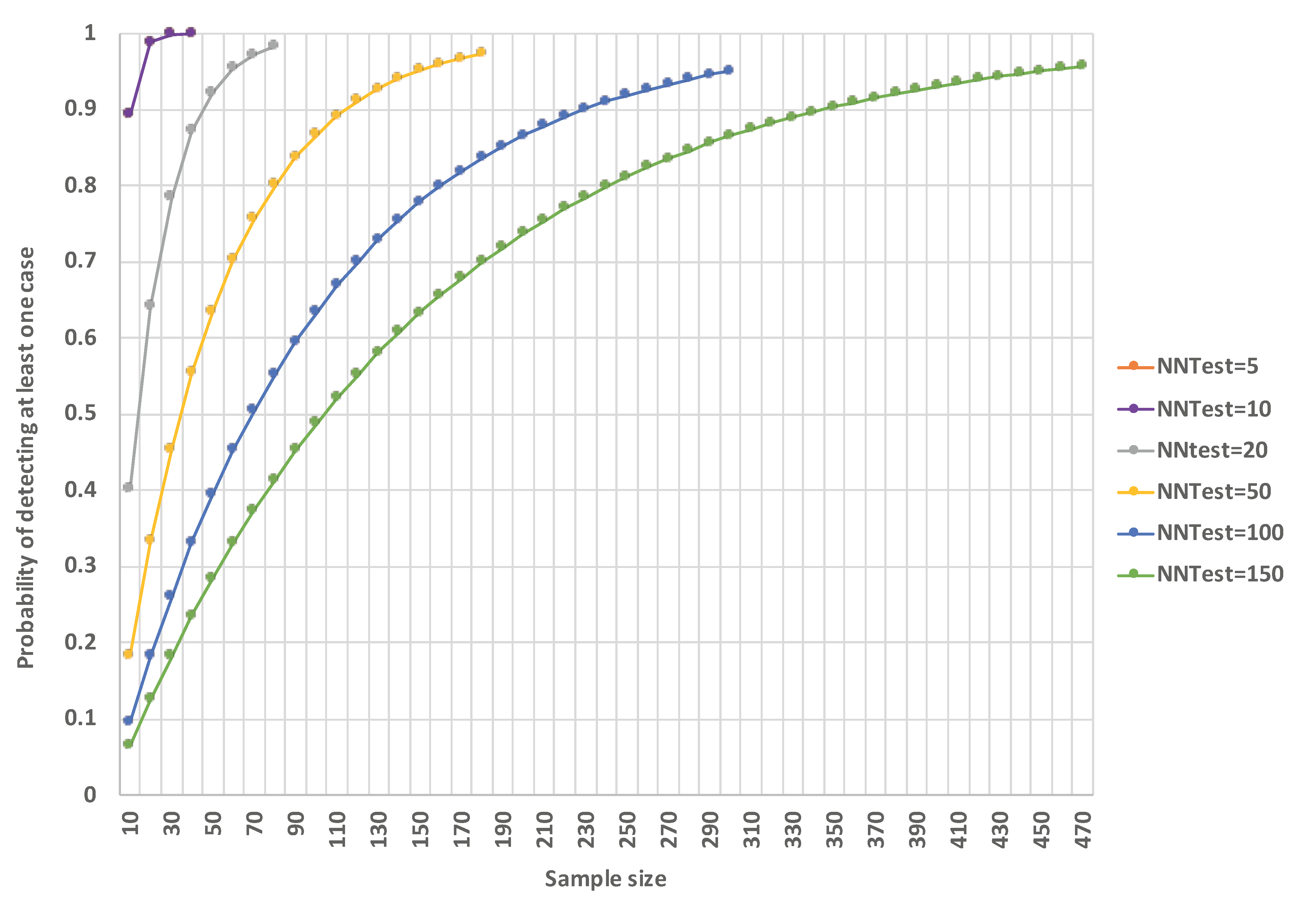

Supplement: S1 Fig — (TIFF) [file pntd.0008916.s002.tiff]
