# Supplementary material for: Potential strategies for strengthening surveillance of lymphatic filariasis in American Samoa after mass drug administration: Reducing ‘number needed to test’ by targeting older age groups, hotspots, and household members of infected persons
Source: PLoS Negl Trop Dis. 2020 Dec 28;14(12):e0008916. doi: 10.1371/journal.pntd.0008916 (PMC7872281; doi:10.1371/journal.pntd.0008916)
Supplement: S1 Table — (DOCX) [file pntd.0008916.s003.docx]

**S1 Table. Probability of selection and post-stratification weights of different subgroups**

|  | **Subgroups** | **Probability of selection** |
| --- | --- | --- |
| Sex | Male  Female | 0.5  0.5 |
| Villages | Hotspots  Index villages  Randomly selected villages | 1.0  1.0  0.43 (30/70) |
| Households | Of index children  In randomly selected villages | 1.0  0.29 |
| Schools | (All elementary schools included) | 1 |
|  | | |
|  | **Age groups (years)** | **Post-stratification weights** |
| Male | 6-7  8-9  10-19  20-29  30-39  40-49  50-59  60-69  70+ | 1  0.91  0.90  1.34  1.36  1.19  0.77  0.74  0.61 |
| Female | 6-7  8-9  10-19  20-29  30-39  40-49  50-59  60-69  70+ | 1  1.07  1.01  0.95  1.19  1.10  0.91  0.74  0.75 |
