# Supplementary material for: Potential strategies for strengthening surveillance of lymphatic filariasis in American Samoa after mass drug administration: Reducing ‘number needed to test’ by targeting older age groups, hotspots, and household members of infected persons
Source: PLoS Negl Trop Dis. 2020 Dec 28;14(12):e0008916. doi: 10.1371/journal.pntd.0008916 (PMC7872281; doi:10.1371/journal.pntd.0008916)
Supplement: S2 Table — (DOCX) [file pntd.0008916.s004.docx]

**S2 Table. Adjustments used for different subgroups**

| **Prevalence estimates for** | **Sex** | **Age** | **Villages** | **Households** | **Schools** |
| --- | --- | --- | --- | --- | --- |
| TAS-3 (6-7year old) | Yes | No | No | No | Yes |
| Community survey of randomly selected villages (≥8 year old) | Yes | Yes | Yes | Yes | No |
| Hotspots | Yes | Yes | No | Yes | No |
| Index villages | Yes | Yes | No | No | No |
| Index households | Yes | Yes | No | No | No |
