# Supplementary material for: Potential strategies for strengthening surveillance of lymphatic filariasis in American Samoa after mass drug administration: Reducing ‘number needed to test’ by targeting older age groups, hotspots, and household members of infected persons
Source: PLoS Negl Trop Dis. 2020 Dec 28;14(12):e0008916. doi: 10.1371/journal.pntd.0008916 (PMC7872281; doi:10.1371/journal.pntd.0008916)
Supplement: S1 Text — (DOCX) [file pntd.0008916.s005.docx]

**S1 Text. Methods used to calculate seroprevalence adjusted for age, sex, and survey design**

Seroprevalence for Ag and antibodies were adjusted for age, sex, and survey design using previously described methods (5) and summarised below. The probabilities of selection and post-stratification weights of different subgroups are summarised in S2 Table, and the adjustments for each subgroup are summarised in S3 Table.

For the school-based survey (TAS-3), we calculated sampling weight for each participant by adjusting for response rates by schools, and applied post-stratification weights for sex to the entire sample.^1^ Community survey estimates accounted for multi-stage cluster sampling design of the survey using the ‘svyset’ command in Stata 13 (StataCorp, 24 College Station, TX). For the randomly selected villages, we calculated a sampling weight for an individual as the inverse product of the probability of selection of the PSU, household and individual. Weights were adjusted according to participation rates within households, and for the proportion of selected households sampled in each village to account for those households which could not be surveyed either due to logistical reasons, non-response or were vacant at the time of village visits. As 30 out of the 70 eligible PSUs were selected, and selection was done without replacement, we applied a finite population correction (FPC) factor of 30/70.^2^

To estimate the country- and village-level Ag and antibody prevalence for people aged ≥8 years, we applied post-stratification weights for age and sex based on American Samoa’s demographic distribution using information available from the 2014 American Samoa Statistical Yearbook (26). Due to the small number of households and participants in some of the randomly selected villages, we combined the following three groups of villages for analyses based on their geographical proximity: i) Vatia, Afono, and Masausi; ii) Asili, Nua, Utumea West, Setaga, and Fagamalo; and iii) Fatumafuti and Faganeanea.

For the hotspot villages of Fagali’i, and Ili’ili,/Vaitogi,/Futiga, the probability of selection of the PSU was set as one because they were purposively selected for the survey. For analyses of index households, index communities, and older children living in index communities, we applied post-stratification weights for age and sex as above. Analyses for these sub-groups were not adjusted by survey design as identification and selection of participants was purposive. For children aged 6-7 years living in hotspot villages of Fagali’i and Ili’ili, Vaitogi, Futiga, we applied post-stratification weight for sex to the sample.

^1^ Sheel M, Sheridan, S, Gass, K, Won, K, Fuimaono, S, Kirk, M, Gonzales, A, Hedtke, SM, Graves, PM, Lau, CL. Identifying Residual Transmission of Lymphatic Filariasis after Mass Drug Administration: Comparing School-Based Versus Community-Based Surveillance - American Samoa, 2016. PLoS Negl Trop Dis. 2018;12(7):e0006583.

^2^ World Health Organization. Vaccination Coverage Cluster Surveys: Reference Manual 2015. Available from: <http://www.who.int/immunization/monitoring_surveillance/Vaccination_coverage_cluster_survey_with_annexes.pdf>
